# Integration of population genetics with oceanographic models reveals strong connectivity among coral reefs across Seychelles

**DOI:** 10.1038/s41598-024-55459-x

**Published:** 2024-03-12

**Authors:** April J. Burt, Noam Vogt-Vincent, Helen Johnson, Ashley Sendell-Price, Steve Kelly, Sonya M. Clegg, Catherine Head, Nancy Bunbury, Frauke Fleischer-Dogley, Marie-May Jeremie, Nasreen Khan, Richard Baxter, Gilberte Gendron, Christophe Mason-Parker, Rowana Walton, Lindsay A. Turnbull

**Affiliations:** 1 Department of Biology, University of Oxford, Oxford, OX1 3SZ UK; 2 Seychelles Islands Foundation, Mont Fleuri, Mahé Seychelles; 3 Department of Earth Sciences, University of Oxford, South Parks Rd, Oxford, OX1 3AN UK; 4 Institute of Zoology, Zoological Society of London, London, NW1 4RY UK; 5 Centre for Ecology and Conservation, University of Exeter, Cornwall Campus, Penryn, TR10 9FE UK; 6 Ministry of Agriculture, Climate Change and Environment, Victoria, Seychelles; 7 Island Conservation Society Seychelles, Pointe Larue, Mahé Seychelles; 8 Island Biodiversity and Conservation Centre, University of Seychelles, Victoria, Seychelles; 9 Marine Conservation Society Seychelles, Transvaal House, Beau Vallon, Mahé Seychelles; 10 Nekton Foundation, Oxford, UK

**Keywords:** Marine biology, Physical oceanography, Population genetics

## Abstract

Many countries with tropical reef systems face hard choices preserving coral reefs in the face of climate change on limited budgets. One approach to maximising regional reef resilience is targeting management efforts and resources at reefs that export large numbers of larvae to other reefs. However, this requires reef connectivity to be quantified. To map coral connectivity in the Seychelles reef system we carried out a population genomic study of the *Porites lutea* species complex using 241 sequenced colonies from multiple islands. To identify oceanographic drivers of this connectivity and quantify variability, we further used a 2 km resolution regional ocean simulation coupled with a larval dispersal model to predict the flow of coral larvae between reef sites. Patterns of admixture and gene flow are broadly supported by model predictions, but the realised connectivity is greater than that predicted from model simulations. Both methods detected a biogeographic dispersal barrier between the Inner and Outer Islands of Seychelles. However, this barrier is permeable and substantial larval transport is possible across Seychelles, particularly for one of two putative species found in our genomic study. The broad agreement between predicted connectivity and observed genetic patterns supports the use of such larval dispersal simulations in reef system management in Seychelles and the wider region.

## Introduction

Coral bleaching is the greatest threat to the persistence of tropical reef ecosystems^1^ and bleaching events are increasing in both frequency and severity^2^. At a global scale, reducing greenhouse gas emissions is the only meaningful way to ensure a future for coral reefs^3^; however, global coral cover has declined by half since the 1950s^4^, transforming the functionality of reefs^5^. Countries at the frontline of climate change are compelled to implement urgent measures to preserve the reef functions on which their financial and social wellbeing rely. Actions can be taken at local scales to improve reef health and resilience^6–9^ but, to maximise their effectiveness, we need better knowledge of the regional reef system in which individual reefs are embedded.

**Figure 1:**
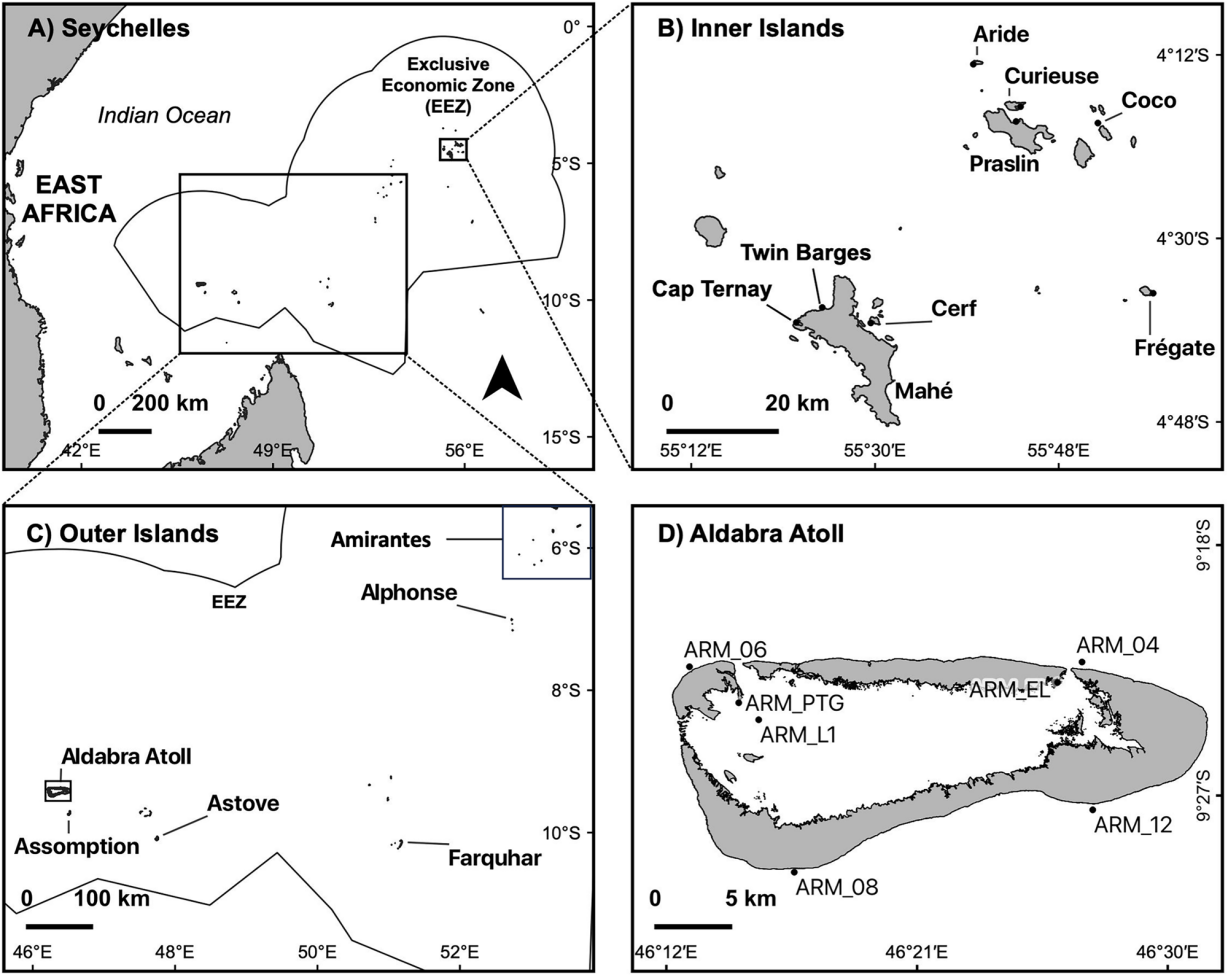
Map of the Seychelles Archipelago (**a**) showing sampling sites in the Inner Islands (**b**), the Outer Islands (**c**) and at Aldabra Atoll (**d**). Site name codes for Aldabra refer to specific Aldabra Reef Monitoring (ARM) locations that were used to collect samples from. Inside the lagoon sites include Point Tanguin (ARM_PTG), Lagoon site 1 (ARM_L1) and East lagoon (ARM_EL).

The Seychelles archipelago is a Small Island Developing State that hosts 13% of the Western Indian Ocean region’s coral reefs^10^ spread across 1.4 million km^2^ of ocean (Fig. 1a). The main inhabited islands in the northern Seychelles are granitic, known as the Inner Islands, situated on the Mahé plateau. To the south-west, there is a smaller group of coralline islands known as the Amirantes, while the Outer Islands in the south include the Aldabra and Farquhar groups. These Outer Islands are also coralline in origin and include several atolls, the largest being Aldabra Atoll, a UNESCO World Heritage Site (Fig. 1d). The entire reef system has already been heavily impacted by coral bleaching^10,11^ and projections for its long-term survival are bleak, with bleaching predicted to become an annual event within the next 15 to 40 years^12^. Mitigation efforts, such as reef restoration and marine spatial planning, are already underway in Seychelles, but to maximise their effectiveness, these efforts need to be properly directed, and this requires better understanding of the connections between reefs.

Broadcast-spawning corals generate enormous numbers of larvae, and only a small number of successful dispersal events are required to establish genetic connectivity between populations^13^. Quantifying the degree of connectivity within a coral metapopulation requires the use of population genetics^14–16^, but oceanographic models that simulate larval dispersal can provide a first-order approximation of whether or not genetic connectivity is likely^17–19^. Both methods have their limitations: genetic studies are expensive and require samples of a single species to be collected across the regional reef system, whereas most oceanographic models do not fully capture the range of physical and biological processes affecting larval dispersal, and rely on poorly constrained parameterisations for larval behaviour^20^.

A recent oceanographic model for the Western Indian Ocean predicts negligible connectivity between Aldabra Atoll and the Seychelles’ Inner Islands^19^. This result matters, because the Outer Islands have been less impacted by bleaching^21^ and may be an important source of resistant larvae for the Inner Islands, which this study appears to rule out. However, due to the relatively coarse spatial resolution of the model and the basic parameterisation for larval behaviour, it is unclear to what extent these findings reflect actual connectivity.

To investigate coral reef connectivity in the Seychelles’ reef system we combined predictions from a fine-scale oceanographic model with direct measurements of genetic connectivity in the broadcast-spawning coral, *Porites lutea*. We chose this species because corals belonging to the *Porites* genus are among the most resilient to bleaching stress^22,23^ and have become dominant on many reefs in Seychelles since the 1998 mass bleaching event. We used genome-wide SNP analysis to estimate: (1) the degree of genetic admixture between the Inner and Outer islands; and (2) the extent of gene flow among reef sites. We then used a 2 km-resolution regional ocean simulation^24^ coupled with a larval dispersal model^25^ to predict the flow of larvae between reef sites. Finally, we determined whether the empirical and modelling approaches returned congruent information about population connectivity, and in doing so, assessed whether oceanographic modelling studies are a robust compliment to genomic studies for large-scale coral reef connectivity mapping.

## Results

### Sequencing and bioinformatics

**Figure 2:**
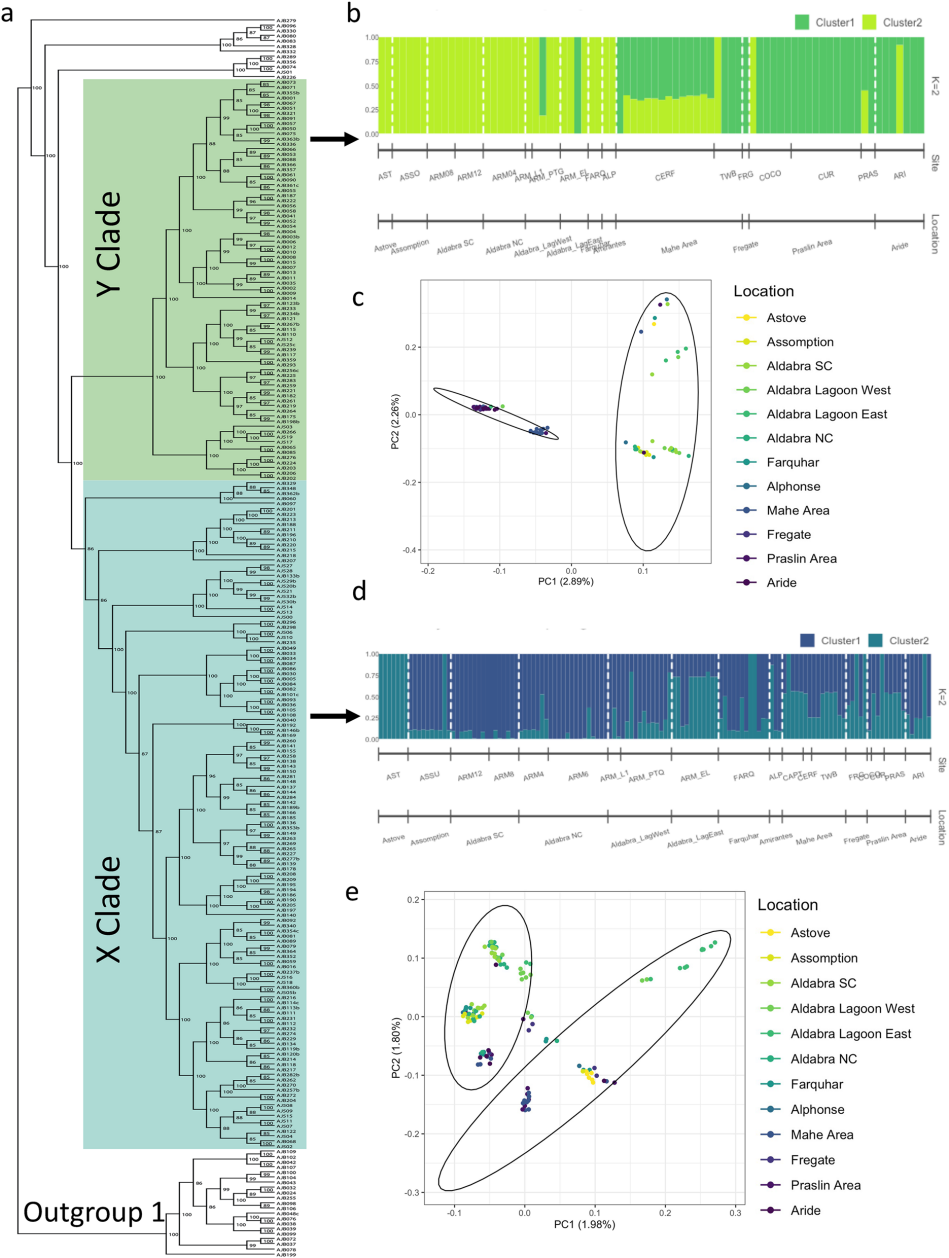
Genetic structure of Porites spp. around Seychelles. (**a**) Phylogenomic relationships of 241 samples (based on 182,511 LD-filtered SNPs) with ultrafast bootstrap support values shown at the internal nodes; (**b**) structure plot for the Y clade (area highlighted green on phylogenetic tree; 78 samples); (**c**) PCA of Y clade; (**d**) structure plot for the X clade (area highlighted blue on phylogenetic tree; 130 samples); (**e**) PCA of X clade. Sites are coloured lightest in the southern (outer) Seychelles, darkest in the northern (inner) Seychelles. Ellipses on PCA figures represent the clusters assigned by admixture analysis [as per (**b,d**)].

We collected a total of 252 samples from individual colonies of *Porites cf. lutea* across the Seychelles archipelago (Table S1). Of these, 241 samples were sequenced and mapped to the 552,020,673 bp *Porites lutea* reference genome^26^ (accession: PRJNA545004; see Methods). The mean mapping rate was 87%, the mean sequencing error rate was 0.02%, the read quality was high (Phred ≥ 30) and the average read depth was 10×. There were 120,538,781 raw variants, which were then filtered for a variety of quality measures, producing a subset of 182,511 SNPs for downstream genetic analyses. Based on the phylogenetic distribution of the full sample set (Fig. 2a), 21 samples had ≥ 5% sequence divergence from the reference genome (Figure S1a) and were therefore likely another species of *Porites*. These samples formed an outgroup (Outgroup 1; Fig. 2a), from which we were able to root the tree using IQTREE^27^ (version 1.6.12). The remaining 220 samples diverged from the reference genome by 0.7–2.2% (Fig. S1b) and were therefore retained for analysis.

### Genetic population structure

Using the model-based clustering method ADMIXTURE^28^, the maximum likelihood estimation of individual ancestries calculated using the full sample set (220 samples), consistently provided the lowest cross-validation error for K = 2 (mean cross-validation error across runs = 0.237), i.e., the most likely number of sub-populations is two. The principal component analysis (PCA) also grouped samples into two distinct clusters (Fig. S2). The samples in each cluster identified by ADMIXTURE and the PCA corresponded to two clades (X and Y in Fig. 2a). There was no clear geographical structure between the two clades: some samples from a given site were entirely assigned to one cluster, while others were entirely assigned to the other, with just 30% of samples having mixed ancestry. This suggests that it is possible the samples within each cluster may belong to two cryptic species, which is not unusual in corals^15,29^. The sequence divergence values for each sample support this view: the samples within the Y clade diverge from the reference genome by 1.19% (SE 0.005) and in the X clade by 0.82% (SD 0.007), a small but significant difference (Fig S1b; *t* = 33.33, df = 206, P < 0.001). Regardless of species delimitations, their divergence from each other and from the reference genome suggest they should be treated as independent evolutionary lineages.

Consequently, we re-analysed the sample sets from the two clades separately using ADMIXTURE, to see whether there was geographical structure within clades. Hereafter they are referred to as the ‘X clade’ (130 samples) and the ‘Y clade’ (78 samples) as per Fig. 2a. For both clades, the maximum likelihood estimation of individual ancestries calculated with ADMIXTURE, consistently provided the lowest cross-validation error for K = 2 (X clade = 0.200; Y clade = 0.347).

Within the Y clade (Fig. 2b), the geographical structure is clear, with the PCA showing segregation of samples from Inner and Outer Islands as expected based on the first two components that explained 2.89% and 2.26% of the variance, respectively (Fig. 2c). This split is reflected in the admixture analysis (Fig. 2b); only 21% of colonies have mixed ancestry. The majority of admixed samples were recovered from Cerf Island, off the east coast of Mahé in the Inner Islands, where nearly all colonies are admixed to a similar degree (~ 35:65).

Within the X clade (Fig. 2d), the PCA clustering is less clear and there are multiple segregations between sample groups with the first two principal components explaining 1.98% and 1.80% of the variance (Fig. 2e). Samples from the eastern end of the lagoon at Aldabra and some of those from the western end form a clear outgroup. The admixture analysis revealed that 74% of samples had mixed ancestry, indicating a high degree of mixing between the reef sites (Fig. 2d). In the Outer Islands, Assomption and the fringing reefs of Aldabra showed similarly low levels of admixture, while greater admixture was apparent in the Aldabra lagoon samples, especially those from the eastern end of the lagoon. However, samples from Astove (also in the Outer Islands) showed no admixture and grouped with samples from the Inner Islands.

**Figure 3:**
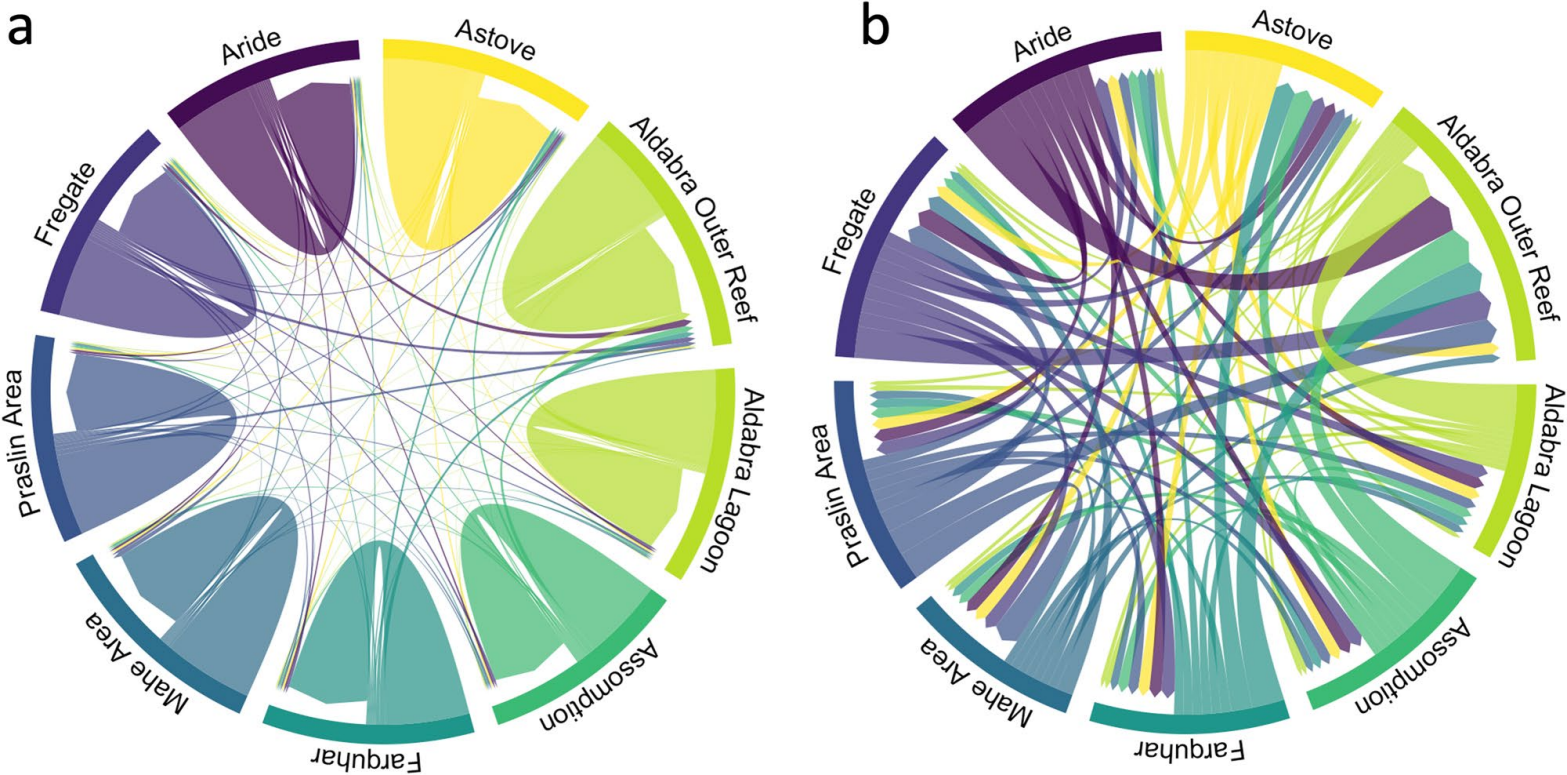
Contemporary gene flow estimates (m) were derived from BayesAss for the X clade identified in Fig. 2A. Chord diagram showing significant gene flow (**a**) within and between island groups/sites and; (**b**) between island groups only (i.e. minus self-recruitment). Arrow width denotes the relative amount of gene flow.

To estimate the genetic variance between our sites we calculated pairwise *Fst* values using the R package HIERFSTAT^30^ for both the X and Y clades; low *Fst* values were estimated for all combinations (Tables S2 and S3). We evaluated the rate and directionality of recent gene flow between pairs of sites using the software BA3-SNPs v1.1.0^30^, a modification of BayesAss^31^ that can handle large SNP datasets. We ran the analysis on the full 220 samples data and then subsequently for the X and Y clades separately for the reasons stated above (Fig. S9). Here we present only the X clade; this clade being the largest (130 samples) and therefore most robust and because we are reasonably confident that it represents a single species, most likely *Porites lutea*. Analyses revealed that self-recruitment was high at all sites (migration rate 77–84%; Fig. 3A, Table S5) with low but uniform migrant exchange between all sites (migration rate 1.2 < 3%; Fig. 3B).

### Comparisons with oceanographic model predictions

We used surface currents from the multidecadal Western Indian Ocean Simulation, WINDS-M^24^, at a 2 km spatial and 30 min temporal resolution and simulated virtual spawning events every day from 1993 to 2019, advecting particles for up to 120 days using OceanParcels^31,32^. Each particle represented a large number of larvae, which continuously attain and lose competency, settle, and die. Based on a larval dispersal model, SECoW (see Methods), we defined *potential connectivity* between source and destination sites as the probability that a coral larva generated by a spawning event at the source site settles at the destination site (taking into account mortality and competency). Due to an absence of direct measurements of the larval characteristics of *Porites lutea*, we assumed that they have similar competency and mortality characteristics to another stony coral with a similar life history strategy^33^, *Platygyra daedalea*. In the absence of a habitat suitability model for *Porites lutea*, we also necessarily assumed that all sites identified as coral reef^34^ are equally suitable for this species.

Our simulations suggested that there is relatively high mean transport of coral larvae across Seychelles (Fig. S3). Potential connectivity decreased with distance, but can exceed 10^–6^ per spawning event between the Inner and Outer Islands. These dispersal probabilities suggested that there may be significant connectivity across the entire Seychelles archipelago. Based on physical larval dispersal alone, the model indicated that most pairs of coral populations within Seychelles may share a common ancestor within tens of generations (Fig. S4), which is consistent with the relatively low Fst values found between populations (Tables S2 and S3).

**Figure 4:**
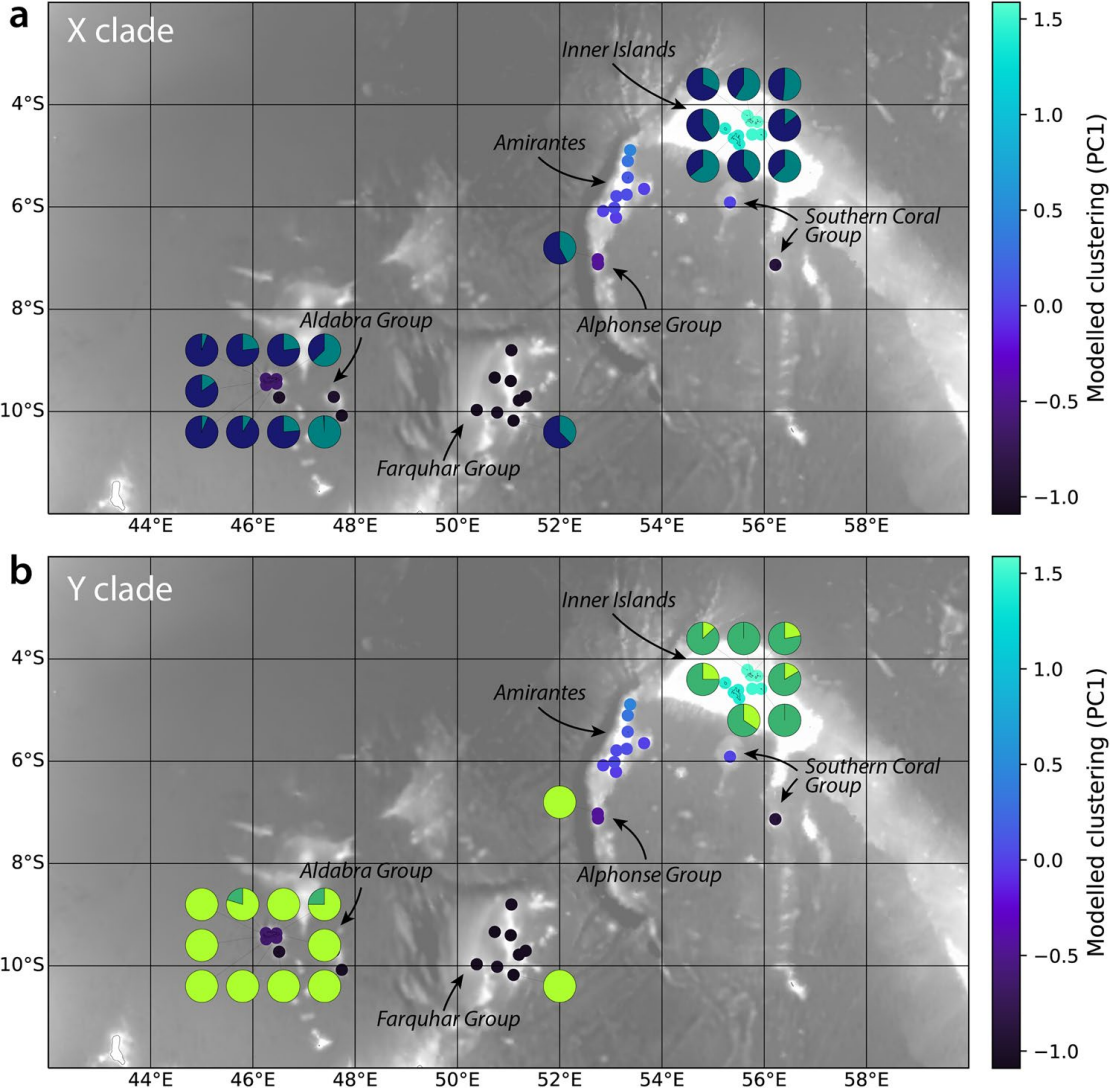
Comparison of the ADMIXTURE analyses for the X (**a**) and Y (**b**) clades, averaged within sampling sites (pie charts). Also shown are simulated clusters of reefs consistently retaining larvae from the oceanographic model (coloured points, where points with similar colours are frequently members of the same simulated cluster).

Despite this high level of connectivity, there are clusters of reefs within Seychelles that tend to retain virtual coral larvae. Although clusters varied through time due to stochastic oceanographic variability, three groupings were relatively robust: (1) the Inner Islands, (2) the Amirante Islands and Southern Coral Group, and (3) the Aldabra and Farquhar Groups. These clusters are shown as coloured points in Fig. 4, where reefs with similar colours tend to exist within the same cluster. Groups 1 and 3 form end-members, with a more consistent dispersal barrier between groups 1 and 2, and a less consistent dispersal barrier between groups 2 and 3 (Figure S5). As a comparison, the pie-charts in Fig. 4 represent the proportions of genetic ADMIXTURE clusters (Fig. 2) for the X and Y clades, averaged within sites.

## Discussion

This study presents the first comprehensive assessment of coral reef connectivity across the Seychelles’ reef system. Our genomic analyses show that in contrast to predictions from previous oceanographic models, genetic admixture does occur between the Inner and Outer Island groups of Seychelles, with contemporary gene flow in both directions. This connectivity is consistent with results from our larval simulations using a higher-resolution oceanographic model, although the realised genetic connectivity revealed by population genomics is greater than that predicted from the model simulations.

Our phylogenomic tree reveals one clear outgroup that may be a different species of *Porites* (possibly *P. lobata*). The remaining samples fall within two main clades (our X and Y clades) and two smaller groups, which vary in their genetic distance from the reference genome. While there has long been evidence that cryptic species of *Porites* occur^35^, delineating species from a divergent population is not straightforward^36^. However, the admixture analyses suggest that the X clade is probably genetically isolated from the Y clade. A similar genetic study using *Porites sp.* to investigate coral connectivity in the Singapore reef system also uncovered three putative cryptic species among a sample of 160 colonies, despite their morphometric analysis suggesting that all samples belonged to one species^15^.

The X and Y clades are each composed of two genetic groups but the geographic structuring of these within-clade groups is quite different. The X clade consists of individuals with a wide range of admixture proportions suggesting that individuals of the two genetic groups freely intermix. Contrastingly, there is obvious geographic structure in the Y clade, indicating a dispersal barrier between inner and outer reefs. The majority of admixture for the Y clade occurs at just one site (Cerf Island) and these are uniformly 35:65 contributions of the two groups; this would be expected in first generation ‘hybridisation’ situations. Assuming that the two genetic clusters within each group formed during some period of reduced gene flow (as required by many models of divergence), then one interpretation of these contrasting patterns is that the two groups within the Y clade represent a relatively recent secondary contact that is in the F1 stage^37^ at the Cerf Island reef. Whether the geographic structure will be maintained will depend on the level of interbreeding and backcrossing between the two groups. The possibility for further cryptic species within this clade could be investigated, and whether this represents an incipient stable contact zone.

The dispersal barrier we detected between the Inner and Outer Islands (distance of > 1000 km between island groups), in both the Y clade and the model simulations is permeable, indicative of significant larval transport. The reasons for the difference in gene flow between X and Y clades are unclear but may be due to differences in the timing of spawning, spawning strategies, habitat suitability, or in larval survival rates among clades^38–40^.

It is notable that estimates of recent gene flow between distant islands inferred from genetic analysis is orders of magnitude higher than the potential connectivity computed from the larval dispersal simulations (Fig. S6). This is surprising, as potential connectivity values revealed by the model should be an upper limit for gene flow, as it assumes no post-settlement mortality or density-dependent death, both of which would tend to further reduce estimated colonisation rates. The discrepancy could be due to incorrect assumptions in one or both datasets. First, we could have underestimated simulated larval dispersal rates in *Porites lutea*. Direct measurements of larval parameters in *Porites lutea* might reveal a higher dispersal capacity than *Platygyra daedalea*, on which the simulations were based; however, further simulations conducted using values from *Acropora valida*^41^, which has lower larval mortality rates and a longer larval competency window, reveal that while potential connectivity may be considerably higher (Fig. S7), it is still significantly lower than observed estimates from genetic analysis. Furthermore, our simulations necessarily assume that larvae generated by corals within lagoons directly enter the open ocean. Together these considerations make underestimations in physical dispersal capacity alone an unlikely explanation for the discrepancy.

Second, the discrepancy may be due to stochastic variability in ocean currents, which can cause short- to mid-term potential connectivity to vary over several orders of magnitude^25^. In this model, such uncertainty is considerable, and can fully explain the mismatch between gene flow and potential connectivity for reefs that are reasonably close together (< 200 km; Fig. S6). However, for more distant reefs, the feasible range of potential connectivity remains significantly and systematically lower than the observed gene flow.

Third, the quality of the inference from BayesAss gene flow analysis increases with increasing numbers of sampled individuals and with the strength of the population structure as measured by F_ST_^42^. The number of samples used in the geographical groupings for our gene flow analysis were varied and reduced in size because of our decision to analyse only the X clade for gene flow. This could therefore have contributed to inflated estimates of gene flow; however, earlier iterations of our analysis where we ran BayesAss using even smaller groupings (individual sampling sites rather than geographical areas; Figure S8) resulted in *lower* estimates of gene flow between sites indicating that our sample size was not inflating gene flow estimates in this case.

Finally, the genetic analysis we used (BayesAss) assumes that the observed genetic structure is due to gene flow between sampled populations, but there are many islands across Seychelles and the wider region that were not sampled in our genetics study. It is therefore also possible that some gene flow between distant populations, assumed by BayesAss to occur through direct dispersal, actually occurs in a stepping-stone manner via sites that were not sampled, such as much of the Amirantes and Farquhar Group or those outside Seychelles. Indeed, the most probable mechanism by which coral larvae from the Outer Islands could reach the Inner Islands is via westwards dispersal towards the east coast of Africa via the East African Coastal Current, where they would then travel north along the coast until reaching the South Equatorial Counter Current^43^ which could bring them eastwards to the Inner Islands. Although this is a long and circuitous route, our models show that such dispersal is a real possibility based on larval competency duration. It is therefore probable that some of the Seychelles Outer Islands, like Aldabra, are a source of larvae for the East African coast, and that, in turn, the East African reefs are a potential source of larvae for the Seychelles Inner Islands, which disperse larvae to the Outer Islands via the southwards Ekman transports, creating a clockwise ‘bus’ route for coral larvae around the region. This potential scenario is consistent with the designation of Seychelles as a biogeographic unit, based on hard coral biogeography across a range of species^44^ and its connection with the wider Western Indian Ocean region.

Considering the patterns of connectivity in terms of reef system management to enhance regional resilience, it is encouraging to think that restoration efforts at any reef along this larval dispersal route could benefit the whole system by supporting or enhancing gene flow via stepping stone connectivity. However, when trying to identify key source reefs within Seychelles to promote local resilience in the face of climate change it is essential to consider connectivity within the three biogeographic groups we have identified; the Outer Islands, Amirantes and Inner Islands. Identifying which reefs within these groups are important source reefs that *most frequently* supply larvae directly to other reefs, and channelling management efforts and resources to these sites would support more effective management of the reef system within Seychelles and the wider region. The qualitative agreement between our predicted connectivity with observed genetic connectivity in patterns, allows us to do this with more confidence.

## Methods

### Genetics

#### Overview

We sampled individual coral colonies from a single putative species across Seychelles. DNA was extracted, sequenced, and mapped against a reference genome. Single nucleotide polymorphisms (SNPs) were called and these were filtered for use in different population genetics analyses to generate measures of population structure based on genetic similarity. One of these, ADMIXTURE, determines the most likely number of populations within the samples set (i.e., the K-value) and the proportion of mixed ancestry (i.e. admixture) for each sample. This was coupled with a Principal Component Analysis (PCA) to likewise assign samples to one population or another. Following this, BAYEASS was used to calculate estimates of geneflow between reef sites and used to identify patterns of connectivity within the Seychelles reef system.

#### Species choice

*Porites lutea* was chosen as the focal species for this study for logistical and biological reasons: (1) it is present and common across WIO reefs; (2) it has a broadcast spawning strategy which is the most common strategy and therefore representative of the broader coral community; (3) the genome for *P. lutea* has already been sequenced^45,46^, allowing us to obtain greater depth of information from samples at the sequencing stage. At collection stage we were aware there are two very morphologically similar species of Porites: *P. lutea* and *P. lobata* in the region and therefore expected that we may end up collecting two species within our sample set.

#### Sample collection

We collected coral fragments from 252 colonies across 19 reef sites (12 different islands; Table S1) in the Seychelles Archipelago from October 2018 to December 2019 (Fig. 1). Samples were imported to the UK under CITES import permit A1160. For each reef site, samples were mostly collected during one SCUBA dive due to logistical constraints. To reduce the chances of sampling clones, colonies were sampled with a distance of at least 5 m between consecutive collections. Sampling was conducted at depths of 5–10 m but most commonly around 7 m at all reef sites except the Aldabra lagoon where shallower reefs meant collection was conducted at around 2 m depth.

#### DNA extraction and quality control

Genomic DNA was extracted using Qiagen DNeasy PowerSoil Pro Kits (Qiagen, Hilden, Germany) with minor tweaks to the manufacturer’s protocol (see Supplementary Material 1) and initial quality was assessed using a Nanodrop^47^. All sample extractions were sent to Novogene UK for further QC testing before sequencing; DNA degradation and contamination were assessed using agarose gel electrophoresis and DNA concentration was measured using Qubit DNA Assay Kit in Qubit 2.0 Flurometer (Life technologies, CA, USA). Of the 152 samples sent, 141 samples were sequenced.

#### Library preparation and sequencing

The library preparation was carried out by Novogene. Genomic DNA was randomly fragmented by sonication to size of ~ 350 bp, and then DNA fragments were end polished, A-tailed, and ligated with the full-length adapters of Illumina sequencing, followed by further PCR amplification with P5 and indexed P7 oligos. The PCR products were purified with AMPure XP system, checked for size distribution by Agilent 2100 Bioanalyzer (Agilent Technologies, CA, USA), and quantified by real-time PCR. The resulting libraries were sequenced on a single Illumina HiSeq4000 lane (Illumina, San Diego, CA, USA) by Novogene UK, using paired-end 150-bp sequencing reads. Samples were processed randomly (not in order of reef site) to avoid plate effects.

#### Read quality control

FASTQ^48^ was used to assess read quality. For each sample Phred score averaged 30–40. The mean error rate for reads was < 0.03% in all samples and therefore very few reads needed removal. Reads containing uncalled bases and/or bases of low quality were discarded using default quality thresholds: (1) adapter-containing reads; (2) paired reads where uncertain nucleotides (Ns) constituted more than 10% of either read; (3) the paired reads when low quality nucleotides (base quality less than 5, Q ≤ 5) constituted more than 50% of either read.

#### Mapping to genome

Burrows-Wheeler Aligner^49^ was utilized to map filtered paired-end reads to the *Porites lutea* genome^26^. The Reference genome was downloaded from: http://plut.reefgenomics.org/. Resulting bam files were then sorted and indexed, with duplicated reads removed using SAMtools^50^ Version 1.

#### Removing symbiont/holobiont DNA

It is likely that coral DNA samples also contained DNA from the symbiotic algae and microbiome that live within coral tissue. Due to the difficulties in separating these non-target species during the extraction process, we relied on post-sequencing processes to remove these. Mapping reads to the coral genome and calling SNPs (Single Nucleotide Polymorphisms) from the mapped reads eliminated any sequences of DNA that were not from the coral genome. Furthermore, we also mapped reads to both the symbiont genome and the holobiont genomes^15^ to see what the mapping rate for these were and whether there is potential for additional analysis specific to symbiont diversity. As expected, the mapping rate to the *P. lutea* genome was highest (55–92%), followed by the symbiont genome (9–10%) and the holobiont genomes (1–2%).

#### SNP calling and filtering

GATK^51^ was used to call SNPs from BAM files and ANNOVAR to annotate variants. The package VCFtools^52^ was used to filter SNPs. We started with 120,538,781 SNPs, we then filtered out variants from contigs less than 10,000 bp long (48,931 SNPs removed). We then filtered to retain only biallelic SNPs and removed SNPs not genotyped in all individuals (12,924,391 SNPs retained). We then converted all sites with less than five reads to missing data and then removed SNPs if > 50% of samples have recorded it as missing data (7,135,042 SNPs retained). None of our samples had over 50% missing data so we retained all for further analysis. Finally, as both the ADMIXTURE package and BayesASS recommends avoiding SNPs with high linkage disequilibrium, we used the ‘–indep-pairwise 100 kb 1 0’ command in PLINK^53^ to remove one of every pair of SNPs with r2 > 0.1 within 100 kb sliding windows (182,511 SNPs were retained).

#### Species verification

We used IQTREE^27^ (version 1.6.12) to produce a phylogenetic tree from the SNP data of the 241 samples (Fig. 2a). The best fitting model of evolution was inferred from the data using IQTREE’s automatic model testing algorithm. A maximum-likelihood phylogenetic tree was then inferred from the alignment and the best-fit model using IQ-TREE’s ultrafast bootstrapping method with 1000 replicates. Specifically, this model was determined to be GTR2 + FO + G4 + ASC (i.e., the general time reversible substitution model for binary data with state frequencies optimized by maximum-likelihood from the data, four discrete Gamma categories of rate heterogeneity and correction for ascertainment bias). The resulting tree was then rooted manually on the monophyletic group of 21 samples that had ≥ 5% sequence divergence from the reference genome because we did not have sequence data from a known outgroup. These divergent samples were removed from further analysis. We checked samples with more than 20% missing/ambiguous data were not clumped together in the tree. As this was not the case we retained those samples for analysis.

#### Population structure analysis

To investigate genetic structure among samples we conducted a Principal Component Analysis (PCA) using the filtered SNP dataset (182,511 SNPs) in the R package SNPRelate^54^. We also examined patterns of population structure by performing maximum likelihood estimation of individual admixture proportions using the program ADMIXTURE^28^, testing K values 1–6. For each value of K, we conducted 100 independent runs and summarised runs using CLUMPP v.1.1.2^55^.

#### Gene flow analysis

We evaluated rates and directionality of recent migration using the software BA3-SNPs v1.1.0^56^, a modification of BayesAss^57^ that allows handling of large SNP datasets. We first assessed the optimal mixing parameters for migration rates (for each sample set), allele frequencies, and inbreeding coefficients by running ten repetitions in BA3-SNP-autotune V 3.0.4 as recommended by Mussmann et al. (2019)^56^. Subsequently, 10 runs of BA3-SNPs were performed using the optimised parameters, 10^7^ iterations, a burn-in of 10^6^ and a sampling interval of 100. Convergence of the chains was then validated using Tracer v1.6^58^, and an average of the gene flow estimates across runs was then calculated. For this analysis, samples were grouped not by specific sampling site but by the broader area, to ensure high samples sizes as suggested Meirmans^42^. Specifically, the samples were grouped by island/area except for the Aldabra reefs where samples were grouped as either outer reef or lagoon.

#### Inbreeding coefficients

To avoid collecting samples that were potentially clones we sampled colonies that were over 5 m apart, however there is still a risk that we collected potential clones. To check this, we have calculated the inbreeding coefficient for each sample site/group using BA3-SNPs v1.1.0^56^. A low inbreeding coefficient means a low level of inbreeding. All sites have very low inbreeding coefficients (X clade < 0.001; Y clade < 0.025; Table S5) and as such the absence of clones is verified.

### Larval dispersal model

In line with previous studies^59–61^, we model coral larvae as positively buoyant, otherwise passive particles that drift according to surface currents, as part of the SECoW system^25^. We use surface currents from the multidecadal Western Indian Ocean Simulation, WINDS-M^24^, covering the entire tropical southwest Indian Ocean at a 2 km spatial and 30-min temporal resolution. We simulate virtual spawning events every day from 1993 to 2019 across all coral reefs within 20 m depth identified from 4.5 m resolution satellite imagery^34^, and advect particles for up to 120 days using OceanParcels^31,32^. Each particle represents a large number of larvae, which attain and lose competency, settle, and die according to the following equations^25,41^:

$$\frac{d{L}_{1}}{dt}=-\left(\alpha +{\mu }_{m}\left(t\right)\right){L}_{1}$$

$$\frac{d{L}_{2}}{dt}=\alpha {L}_{1}-\left(\beta +{\mu }_{m}\left(t\right)+{\mu }_{s}{F}_{r}\left(t\right)\right){L}_{2}$$

where ${L}_{1}$ and ${L}_{2}$ respectively refer to the proportion of larvae represented by a particle that are precompetent (1) and competent (2); $\alpha$ is the competency acquisition rate, and is zero before a minimum competency period ${t}_{c}$; $\beta$ is the competency loss rate; ${\mu }_{m}(t)$ is the time-varying mortality rate; ${\mu }_{s}$ is the settling rate; and ${F}_{r}\left(t\right)$ is the proportion of the grid cell occupied by the particle that is covered by coral reef.

We further assume that corals have a constant fecundity $\rho$. We define a ‘settling event’ as the time a larva is within a grid cell where ${F}_{r}>0$ and $t\ge {t}_{c}$. The number of larvae $S$ settling during settling event $j$ is given by:

$${S}_{j}=\frac{{\rho A}_{i}{\mu }_{s}{F}_{r}^{j}}{N}{\int }_{{\tau }_{0}^{j}}^{{\tau }_{0}^{j}+\Delta {\tau }^{j}}{L}_{2}\left(t\right) dt$$

where ${A}_{i}$ is the area of the source reef cell for the particle, $N$ is the number of particles released per cell, ${F}_{r}^{j}$ is the reef fraction of the destination cell, and ${\tau }_{0}^{j}$ and $\Delta {\tau }^{j}$ are respectively the start time and duration of the settling event. By computing ${S}_{j}$ for all particles and settling events (up to a maximum of 60 per particle), we obtain a potential connectivity matrix giving the proportion of larvae transported between reef sites as a function of spawning date.

Analysis of the temporal variability of potential connectivity revealed a significant dependence on the monsoonal cycle, particularly for the outer islands (Figure S3^25^). Since corals in the southwest Indian Ocean tend to spawn between the months of October and March (e.g. ^62,63^), we therefore only considered the subset of virtual spawning events during these months.

As described above, SECoW requires a number of parameters describing larval biology. These parameters have not been measured for *Porites lutea*. Instead, we use parameters for another stony coral, *Platygyra daedalea*^41^, but our main results are relatively insensitive to most of these parameters.

### Clustering

To identify groups of reefs that tend to retain larvae (and therefore, potentially, gene flow), we use the Map Equation via the Infomap algorithm^64^. Based on a directed and weighted graph, Infomap partitions the network represented by the graph into modules by minimising the information required to describe a random walk across that network. In the context of a graph representing dispersal likelihood between pairs of reefs, modules identified by Infomap correspond to collections of reefs that tend to preferentially exchange larvae amongst one another.

However, larval dispersal is highly stochastic and patterns of dispersal may vary significantly depending on the exact timing of dispersal^25,65,66^. To identify clusters of reefs that *consistently* strongly exchange larvae across stochastic oceanographic variability, we assume that spawning occurs between October and March^62,63^, and generate 1000 possible short-term connectivity matrices, each computed as the mean across ten random spawning events. These connectivity matrices are based on reef groups (clusters of reef cells identified through an agglomerative distance-based clustering scheme) rather than individual reef cells to make computation tractable. We then individually partition the 1000 connectivity matrices into modules using Infomap, based on a two-level partitioning and 1% teleportation probability for stability. This results in a length-1000 vector for each of the 38 reef groups in Seychelles in SECoW, which each element corresponding to the module the group has been assigned to for each possible connectivity matrix.

To identify consistent clustering, we pass these vectors through a principal component analysis (PCA). The PCA reveals that 63.8% of the variance in module membership over time is explained by the first principal component, PC1, with 17.2% explained by PC2. Three reef clusters appear with PC1 and PC2 (Fig. S5), broadly corresponding to (1) the Inner Islands, (2) the Amirante Islands and Southern Coral Group, and (3) the Aldabra and Farquhar Groups.

(1) The Aldabra and Farquhar Groups, (2) the Amirante Islands and Southern Coral Group, and (3) the Inner Islands. The distance between pairs of sites in principal component space is related to how *consistently* those sites are assigned to the same module. However, the distinction between clusters (2) and (3) is largely made by PC2, which only explains a relatively small proportion of the variability in module membership compared to PC1. We therefore focus on PC1, with differences in PC1 acting as a proxy for how consistent larval exchange is between pairs of reefs. The value of PC1 is low (< 0.5) within the Aldabra and Farquhar Groups, intermediate within the Amirante Islands and Southern Coral Group (−0.5 to 0.5), and very high within the inner islands (~ 1.5).

### Gene flow

Gene flow between reef sites inferred by BayesAss reflects recent dispersal^67^. To quantitatively compare these predictions to estimates from the oceanographic model, we again generate 1000 possible short-term connectivity matrices from SECoW. Each matrix is computed as the mean across ten randomly chosen spawning events between October and March, thereby sampling the full range of stochastic oceanographic variability. Contrary to clustering analyses using Infomap which require information about the full network structure, only information between sampling sites is required for comparison with BayesAss gene flow estimates. We therefore directly compare modelled estimates of larval flow between pairs of sampled sites from SECoW, to the gene flow estimates from BayesAss. Note that there are considerable uncertainties (1) associated with fitting posterior probabilities to SNP data in BayesAss and (2) introduced by stochastic oceanographic variability.

### Supplementary Information

Supplementary Information.

## Data Availability

The datasets generated and/or analysed during the current study are archived on Zenodo and can be accessed here. The oceanographic model output, WINDS-M, can be downloaded from the CEDA Archive here.
