# Supplementary material for: Integration of population genetics with oceanographic models reveals strong connectivity among coral reefs across Seychelles
Source: Sci Rep. 2024 Mar 12;14:4936. doi: 10.1038/s41598-024-55459-x (PMC10933301; doi:10.1038/s41598-024-55459-x)
Supplement: Supplementary file 1 — Supplementary Information. [file 41598_2024_55459_MOESM1_ESM.pdf]

Integration of population genetics with oceanographic models reveals strong connectivity among coral reefs across Seychelles

April Burt[1,2]*, Noam Vogt-Vincent[3], Helen Johnson[3], Ashley Sendell-Price[1], Steve Kelly[1], Sonya M. Clegg[1], Catherine Head[4], Nancy Bunbury[2,5], Frauke Fleischer-Dogley[2], Marie-May Jeremie[6], Nasreen Khan[7], Richard Baxter[8], Gilberte Gendron[8], Christophe Mason-Parker[9], Rowana Walton[10], Lindsay A Turnbull[1]

Supplementary Figures and Tables

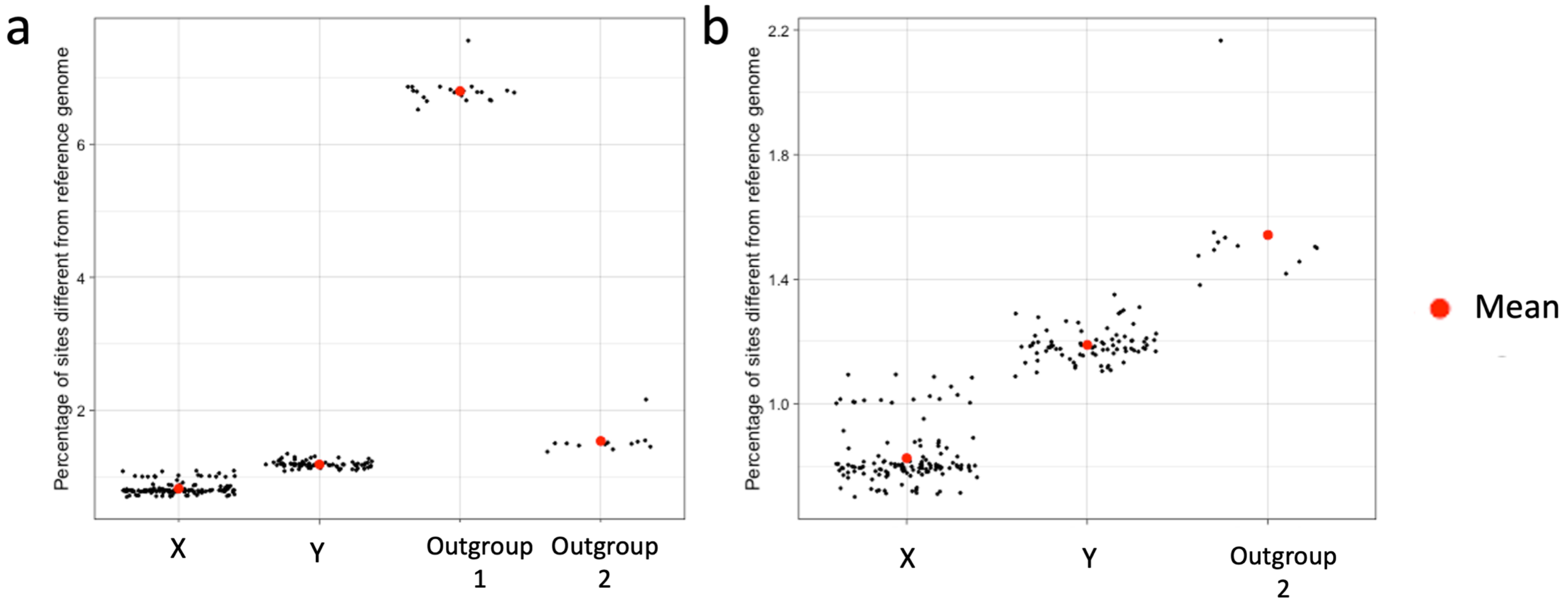

*Figure S1: The percentage of SNPs for each sample that were different from the reference genome, for: a) All 241 samples and b) excluding the main outgroup which has >5% divergence from the reference genome. Samples were grouped based on the phylogenetic clade they fell into in Fig 2a. Calculation for each point: (Total snps - snps that were low coverage or heterozygous)*the number of genome reference sites.*

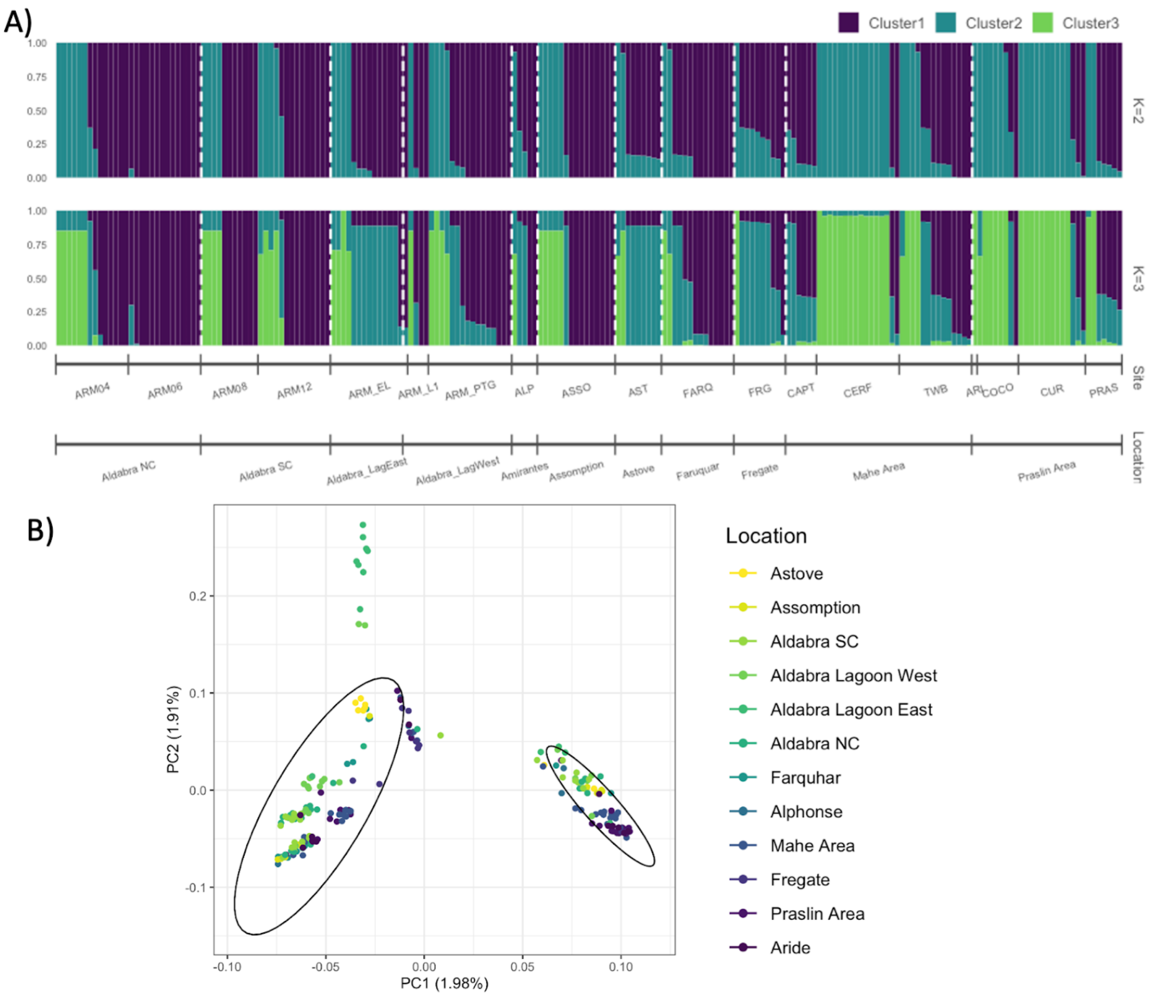

Figure S2: Population structure for the 220 samples (182,511 LD-filtered SNPs) included in initial analysis (excluding the outgroup identified on tree which had a higher divergence (>=5%) to the genome reference than the rest (~1-2% divergence)): A) Admixture plots for K=2 & 3, mean cross-validation error for 100 runs of ADMIXTURE: K2= 0.237; K3= 0.242; B) Principal component analysis of genetic variation in Porites cf. lutea, the variance explained by PC1 and PC2 is 1.98% and 1.11% respectively. Sites are coloured lightest in the south, darkest in the northern Seychelles.

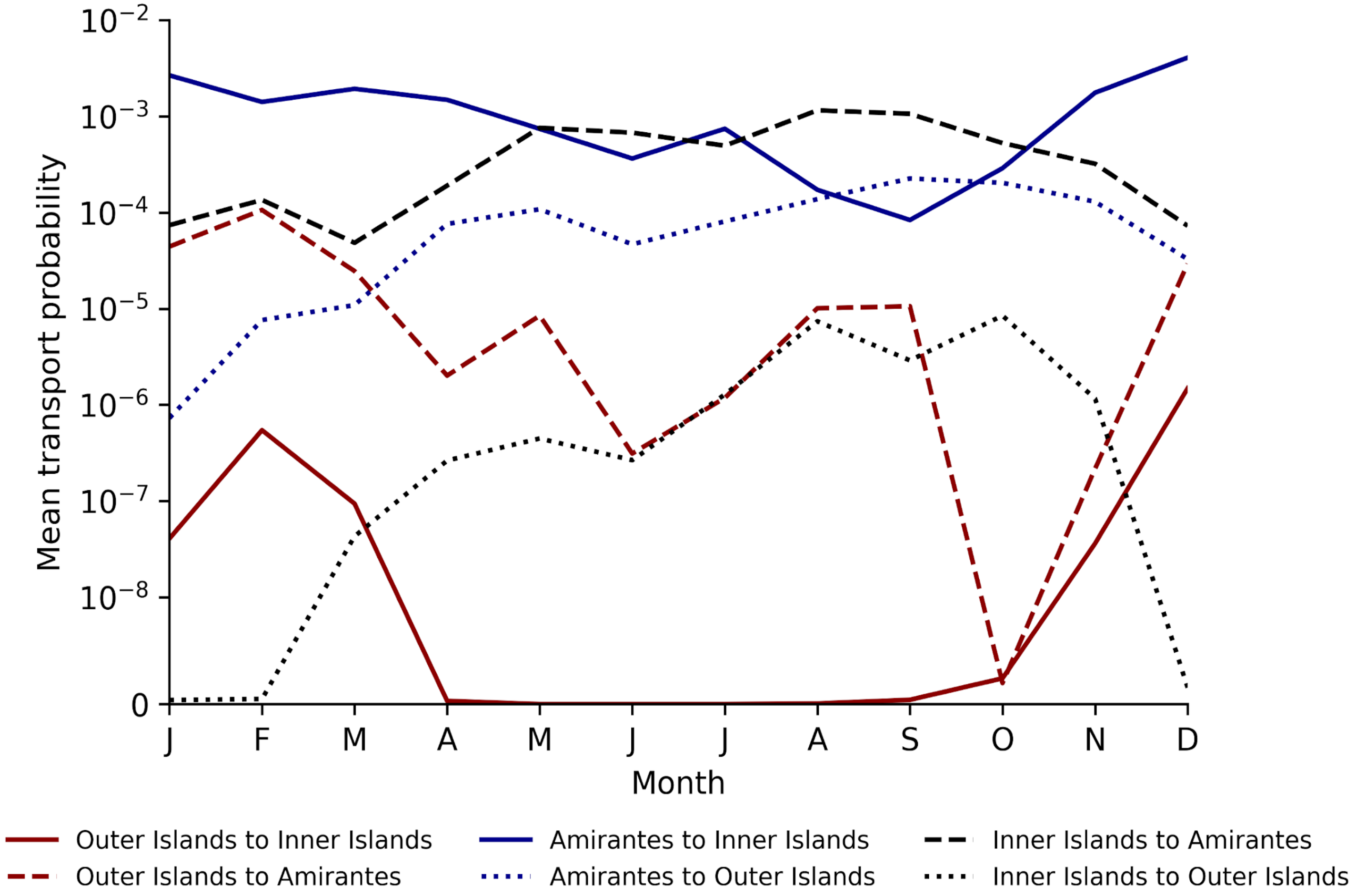

Figure S3: Mean transport probabilities between island groups across the seasonal cycle. Here, we are referring to the Aldabra and Farquhar Groups as the 'Outer Islands'; the Amirante Islands and Southern Coral Group as 'Amirantes', and the islands on the Seychelles Plateau as the 'Inner Islands'.

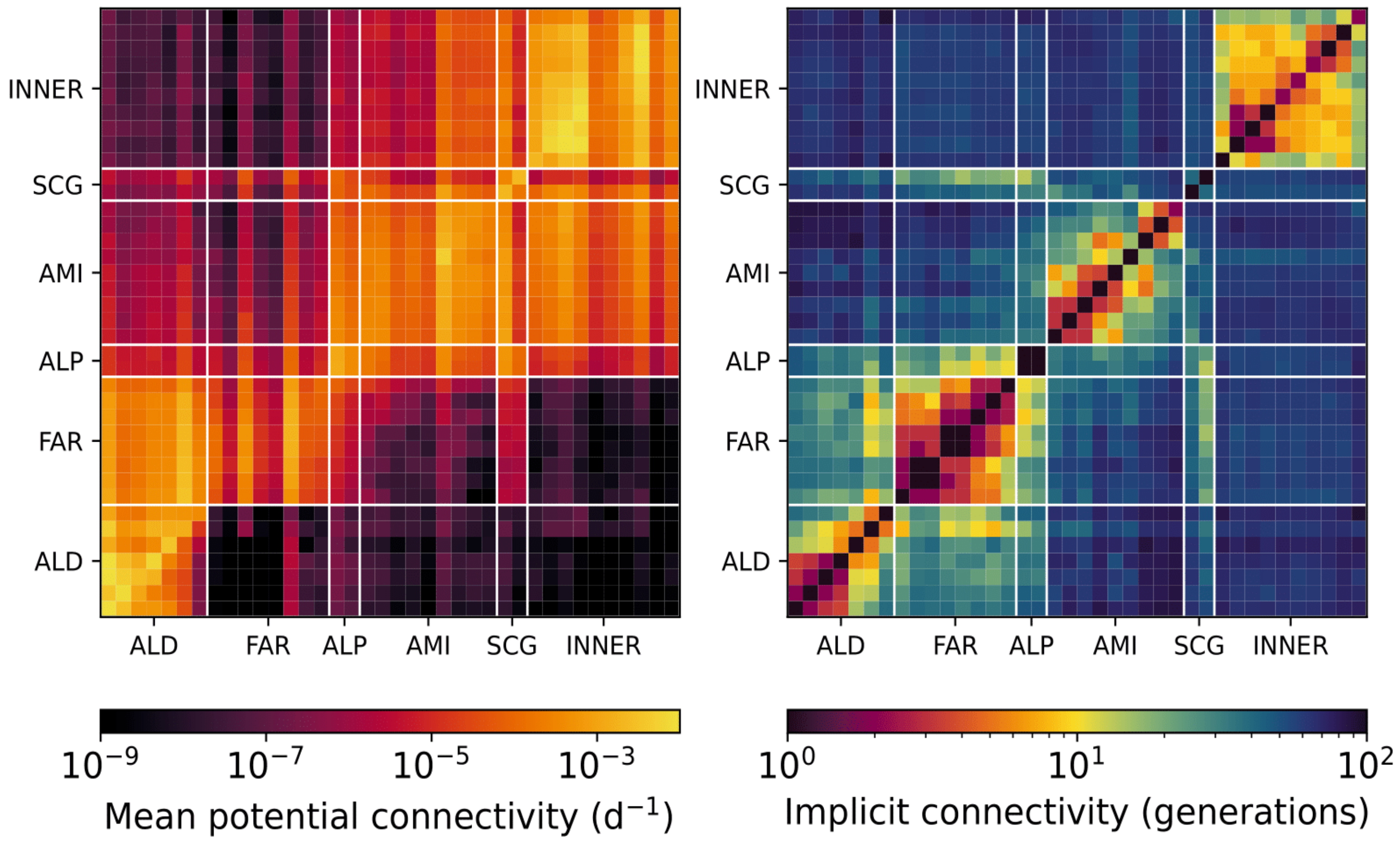

Figure S4: Left*: Time-mean potential connectivity between pairs of reef groups in Seychelles, expressed as transport probability (the* y and *x axes respectively indicate source and destination sites).* Right: *Implicit connectivity between pairs of reef groups in Seychelles, expressed as the median number of generations of dispersal separating pairs of reef groups (i.e. the median number of generations required for the backward cumulated implicit connectivity to exceed 0.5). Labels refer to island groups: Inner (Inner Islands), SCG (Southern Coral Group), AMI (Amirante Islands), ALP (Alphonse Group), FAR (Farquhar Group), and ALD (Aldabra Group).*

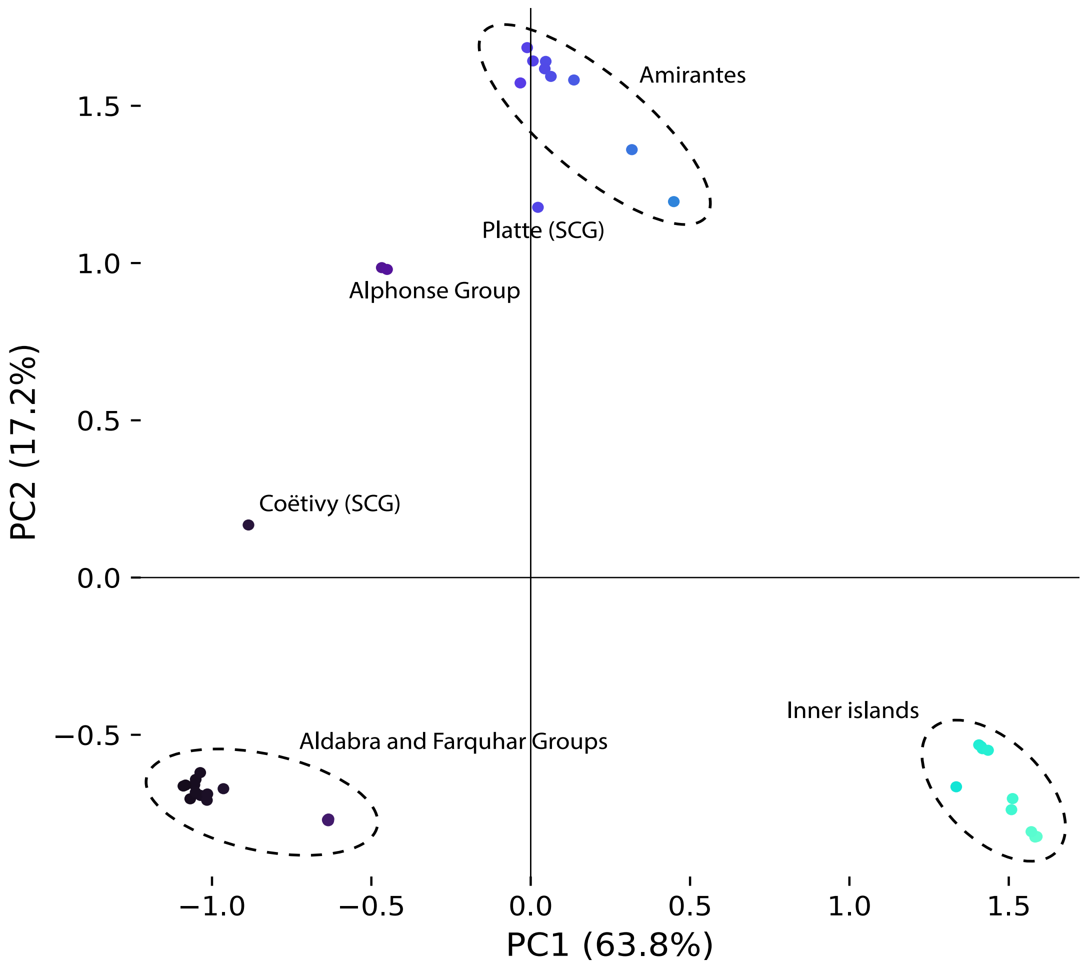

Figure S5: PCA to identify consistent clusters of reefs identified through Infomap. Points represent reef groups, and are coloured according to PC1. Reef groups that cluster together in PC1-PC2 space are frequently identified by Infomap as belonging to the same module.

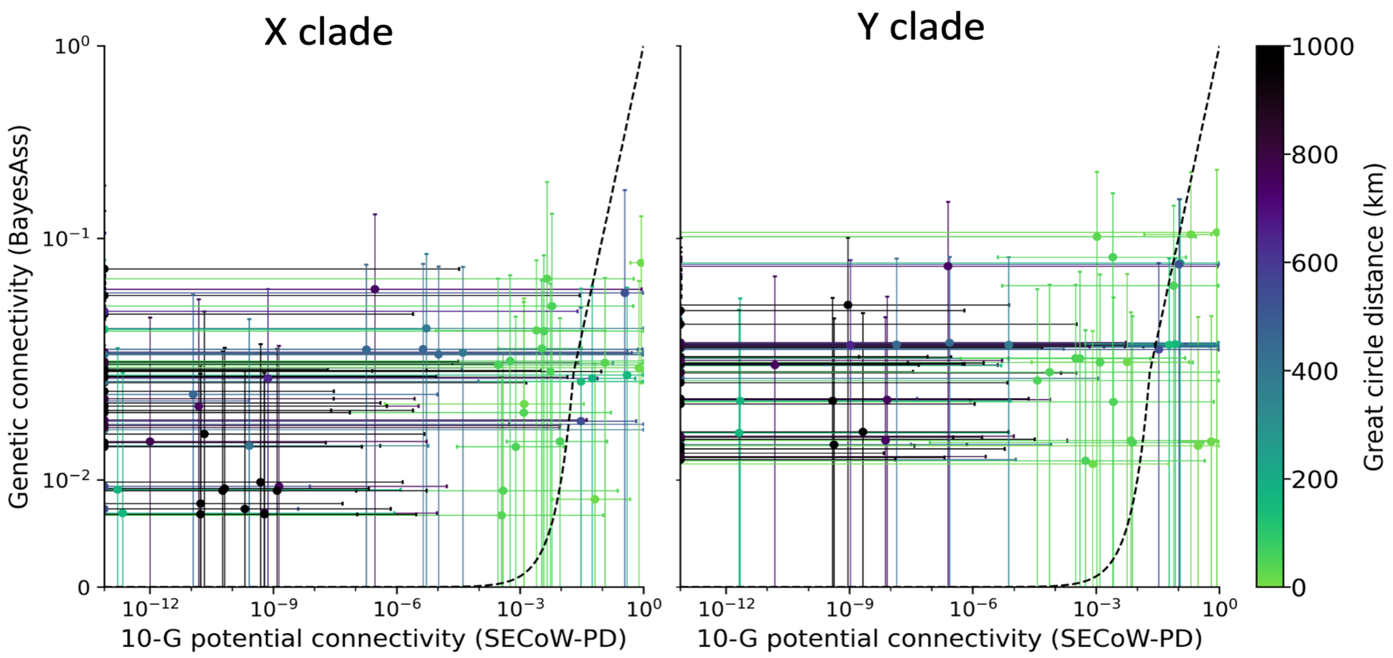

Figure S6: Gene flow inferred by BayesAss plotted against the mean larval flow between corresponding reef sites from SECoW across 10 generations, computed as the median across 1000 possible 10-year connectivity matrices (based on parameters for P daedalea). Error bars reflect the 95% confidence intervals. Note that gene flow is directional, so each pair of reefs is represented by two points. Points are coloured by their great-circle (shortest) distance. Self-recruitment is not plotted. The dotted line represents the 1:1 ratio between connectivity inferred from BayesAss, and potential connectivity computed from SECoW. Note that this line appears non-linear due to the symlog axes with different linear thresholds.

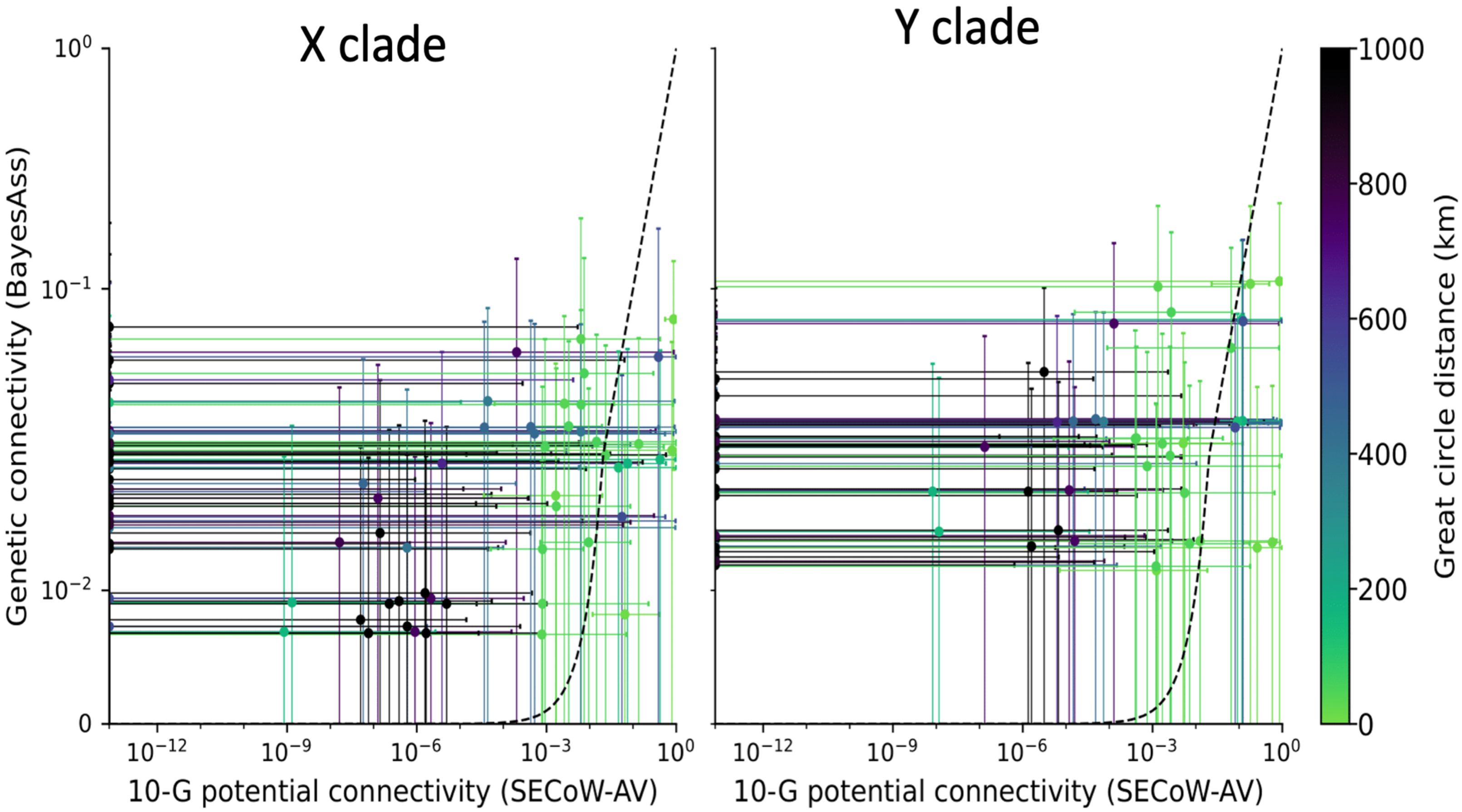

Figure S7: As in Figure S6, but based on parameters for A valida.

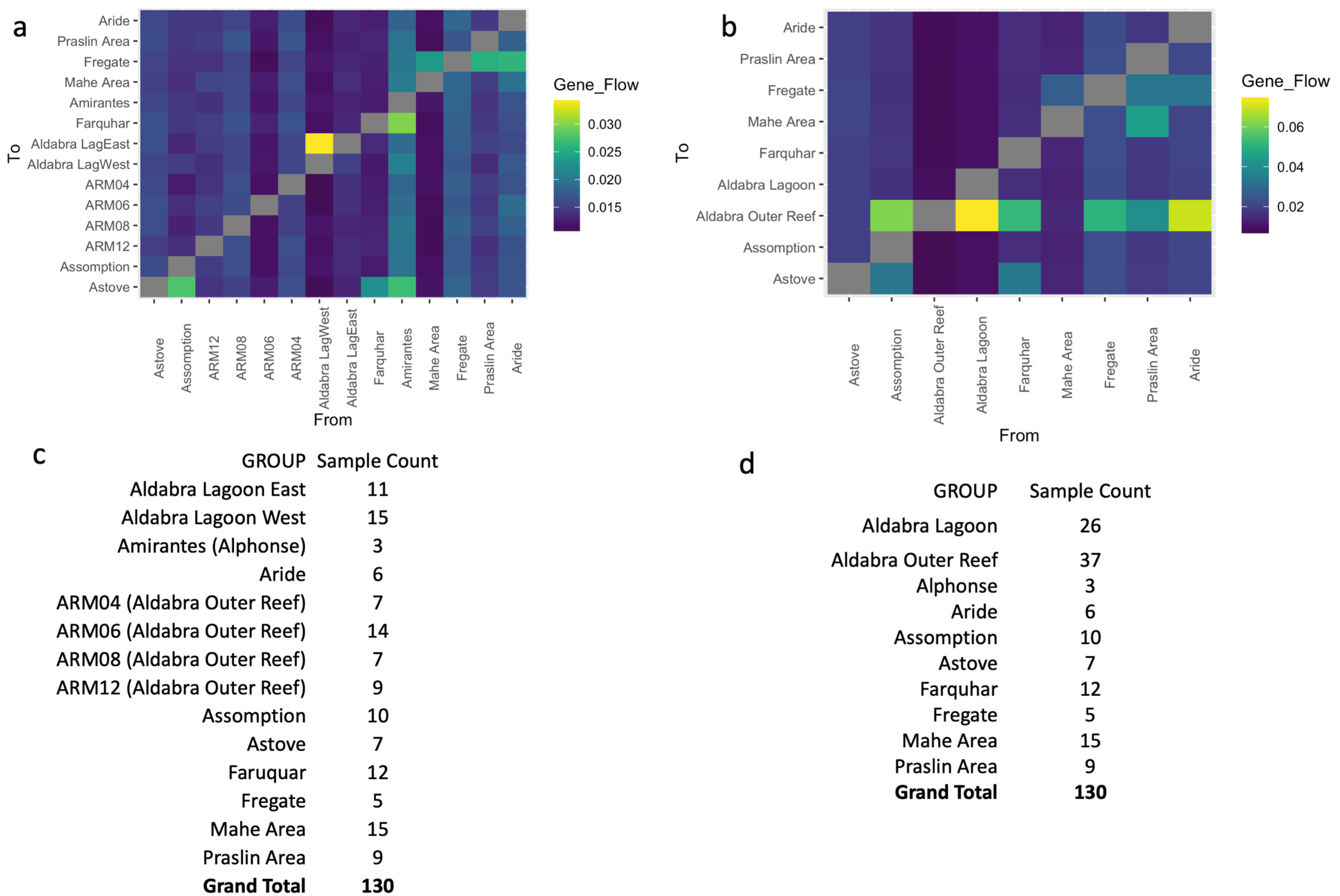

c

GROUP	Sample Count
Aldabra Lagoon East	11
Aldabra Lagoon West	15
Amirantes (Alphonse)	3
Aride	6
ARM04 (Aldabra Outer Reef)	7
ARM06 (Aldabra Outer Reef)	14
ARM08 (Aldabra Outer Reef)	7
ARM12 (Aldabra Outer Reef)	9
Assomption	10
Astove	7
Faruquar	12
Fregate	5
Mahe Area	15
Praslin Area	9
**Grand Total**	**130**

d

GROUP	Sample Count
Aldabra Lagoon	26
Aldabra Outer Reef	37
Alphonse	3
Aride	6
Assomption	10
Astove	7
Farquhar	12
Fregate	5
Mahe Area	15
Praslin Area	9
**Grand Total**	**130**

Figure S8: Comparison of gene flow estimates from BayesAss analysis for the X clade, using the same sample set but with different sized groupings. Original analysis separated the specific sampling sites at Aldabra (a & c) but the results presented in this study use the broader grouping (b & d).

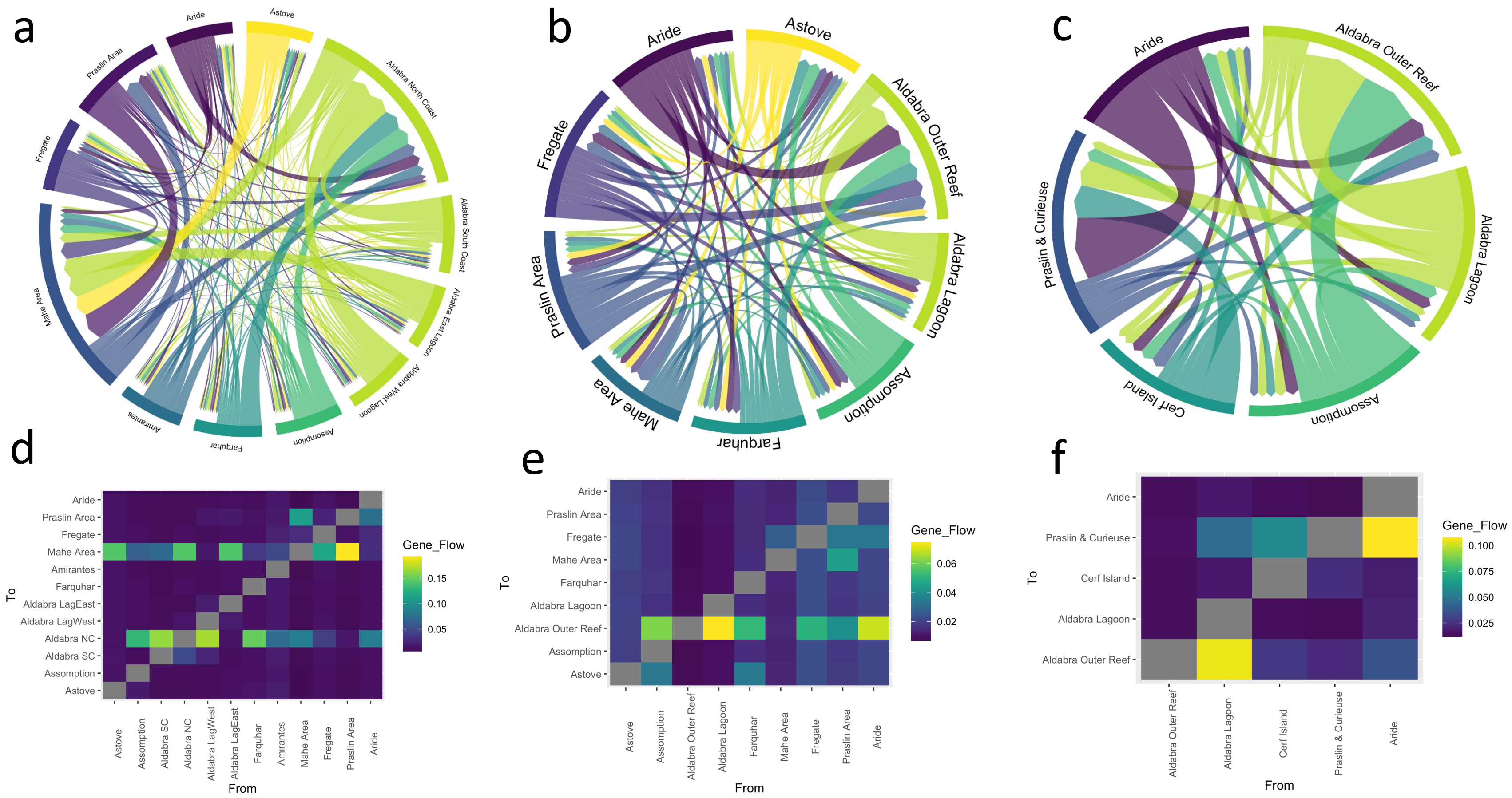

Figure S9: Gene flow estimates from BayesAss analysis for the full 220 sample set (A&D), the X clade (B&E) and the Y clade (C&F). Data for these plots is available [here](#).

Table S1: Sample sites and quantities. 241 samples were sequenced, 21 of these were not included in analysis because they had significant divergence from the reference genome.

Site	Island group/location (& gene flow analysis grouping)	# 220 Samples included in analysis	# Samples in X Clade	# Samples in Y Clade
Aldabra Reef Monitoring Site 6 (ARM06)	Aldabra North Coast	14	14	0
Aldabra Reef Monitoring Site 4 (ARM04)		14	1	6
Aldabra Reef Monitoring Site 8 (ARM08)	Aldabra South Coast	11	7	4
Aldabra Reef Monitoring Site 12 (ARM12)		14	9	4
Aldabra West Lagoon Site 1 (ARM_L1)	Aldabra West Lagoon	4	3	1
Aldabra Point Tanguin West Lagoon (ARM_PTQ)		16	12	4
Aldabra East Lagoon (ARM_EL)	Aldabra East Lagoon	15	11	4
Assomption (ASSU)	Assomption	15	10	5
Astove (AST)	Astove	9	7	2
Farquhar (FARQ)	Farquhar	14	12	2
Alphonse (ALP)	Amirantes	6	3	2
Cap Ternay (CAPT)	Mahé Area	6	5	
Twin barges (TWB)		14	8	4
Cerf Is (CERF)		16	2	14
Fregate (FRG)	Fregate	10	5	1
Praslin APC (PRAS)	Praslin Area	7	5	2
Curieuse (CUR)		13	3	10
Ile Coco (COCO)		8	1	6
Aride (ARI)	Aride Island	14	6	7
TOTAL		220	130	78

Table S2: Pairwise Fst calculated for the X clade using R Package HIEFSTAT (colourmap with green being the relative highest Fst, red being relative lowest)

	AldabraLagoon	AldabraOuterReef	Alphonse	Assomption	Astove	Farquhar	Fregate	MaheArea	PraslinArea	Aride
AldabraLagoon		0.006838291	0.01013218	0.01140641	0.0194967	0.0087449	0.01259096	0.01209355	0.01008049	0.00984631
AldabraOuterReef	0.006838291		0.00157032	0.00436901	0.01754938	0.00345436	0.00868826	0.00844949	0.00637523	0.00441444
Alphonse	0.010132183	0.001570323		0.0014286	0.01340237	-0.001166	0.00773101	0.00749936	0.00427997	0.00186179
Assomption	0.011406413	0.004369008	0.0014286		0.01723225	0.00250144	0.0099887	0.00929876	0.00693801	0.00461096
Astove	0.019496697	0.017549379	0.01340237	0.01723225		0.01212342	0.01794083	0.01762764	0.01639856	0.01693945
Farquhar	0.008744901	0.003454361	-0.001166	0.00250144	0.01212342		0.00576156	0.00552108	0.00381928	0.0013709
Fregate	0.01259096	0.008688263	0.00773101	0.0099887	0.01794083	0.00576156		0.00741829	0.00482459	0.00212132
MaheArea	0.012093552	0.00844949	0.00749936	0.00929876	0.01762764	0.00552108	0.00741829		-0.0005121	0.00256632
PraslinArea	0.010080492	0.006375231	0.00427997	0.00693801	0.01639856	0.00381928	0.00482459	-0.0005121		0.00177242
Aride	0.009846311	0.004414439	0.00186179	0.00461096	0.01693945	0.0013709	0.00212132	0.00256632	0.00177242	

Table S3: Pairwise Fst calculated for the Y clade using R Package HIEFSTAT (colourmap with green being the relative highest Fst, red being relative lowest)

	Aldabra_Lagoon	Aldabra_Outer_Reef	Assomption	Astove	Farquhar	Alphonse	Fregate	Mahe_Area	Praslin_Area	Aride
Aldabra_Lagoon	NA	0.0033	0.0109	0.0023	0.0049	0.0050	0.0094	0.0080	0.0085	0.0088
Aldabra_Outer_Reef	0.0033	NA	0.0041	-0.0003	0.0004	0.0027	0.0094	0.0076	0.0092	0.0081
Assomption	0.0109	0.0041	NA	0.0007	0.0030	0.0054	0.0156	0.0099	0.0121	0.0103
Astove	0.0023	-0.0003	0.0007	NA	-0.0142	-0.0103	0.0005	0.0036	0.0051	0.0045
Farquhar	0.0049	0.0004	0.0030	-0.0142	NA	-0.0099	0.0010	0.0051	0.0063	0.0054
Alphonse	0.0050	0.0027	0.0054	-0.0103	-0.0099	NA	0.0013	0.0044	0.0068	0.0055
Fregate	0.0094	0.0094	0.0156	0.0005	0.0010	0.0013	NA	0.0014	-0.0030	-0.0030
Mahe_Area	0.0080	0.0076	0.0099	0.0036	0.0051	0.0044	0.0014	NA	0.0032	0.0034
Praslin_Area	0.0085	0.0092	0.0121	0.0051	0.0063	0.0068	-0.0030	0.0032	NA	-0.0004
Aride	0.0088	0.0081	0.0103	0.0045	0.0054	0.0055	-0.0030	0.0034	-0.0004	NA

Table S4: Sample groupings for BayesASS analysis based on the 130 blue clade samples only and presented in Figure 3 of this study (Alphonse data not plotted due to low sample size).

**Site/Area grouped**	**Count of sample.id**
Aldabra Lagoon	26
Aldabra Outer Reef	37
Alphonse	3
Aride	6
Assomption	10
Astove	7
Farquhar	12
Fregate	5
Mahé Area	15
Praslin Area	9
**Grand Total**	**130**

Table S5: Inbreeding coefficients for samples within each clade at each sampling location, calculated using BA3-SNPs v1.1.0[240] software.

Site/Group	Fstat X Clade	Fstat Y Clade
Alphonse	0.0014(0.0016)	0.0200(0.0157)
Farquhar	0.0009(0.0019)	0.0083(0.0080)
Astove	0.0000(0.0000)	0.0167(0.0112)
Cerf	NA	0.0023(0.0065)
Mahé Area	0.0000(0.0000)	NA
TwinBarges	NA	0.0069(0.0055)
Praslin Area	0.0010(0.0019)	0.0000(0.0000)
Assomption	0.0004(0.0008)	0.0065(0.0077)
Aldabra Outer Reef	0.0000(0.0000)	0.0000(0.0000)
Aldabra Lagoon	0.0000(0.0000)	0.0067(0.0076)
Fregate	0.0004(0.0010)	NA
Aride	0.0005(0.0008)	0.0042(0.0039)
